# The influence of academic values on academic engagement among Chinese college students: A serial mediation model

**DOI:** 10.1371/journal.pone.0350617

**Published:** 2026-06-24

**Authors:** Jingyi Dong, Tiantian Yang, Zhixia Wei, Xiaoxia Gu, Yangshu Ru, Ling Hu

**Affiliations:** 1 Faculty of Educational Studies, Universiti Putra Malaysia, Selangor, Malaysia; 2 Office of International and Exchange, Yunnan Minzu University, Chenggong, Kunming Yunnan, China; 3 Hebei Key Laboratory of Children’s Cognition and Digital Education, School of Educational Studies, Langfang Normal University, Langfang, Hebei Province, China; 4 School of Public Administration, Nanfang College Guangzhou, Guangzhou, China; 5 Guangxi Minzu University, East Road, Xixiangtang District University, Nanning, Guangxi Zhuang Autonomous Region, China; 6 School of Economics, Sichuan University, Chengdu, China; Tianjin University, CHINA

## Abstract

This study investigated how academic values shape academic engagement among Chinese college students by examining the mediating roles of academic self-efficacy, autonomous motivation, and controlled motivation. Using a quantitative design with random sampling, valid data were collected from 1,110 students who completed four validated instruments: the College Students’ Academic Values Questionnaire, the Academic Self-Efficacy Scale, the Academic Motivation Questionnaire, and the Academic Engagement Scale. Structural equation modeling was used to test a serial mediation model. The findings revealed that academic self-efficacy, autonomous motivation, and controlled motivation mediated the relationship between academic values and academic engagement. Moreover, academic self-efficacy and both motivational types formed significant serial mediation pathways that explain how values influence engagement. Notably, the mediation pathways involving controlled motivation produced negative indirect effects, suggesting that controlled motivation may weaken students’ academic engagement.

## 1. Introduction

Academic engagement is widely regarded as a key driving force in college students’ educational experience, shaping not only their academic performance but also their personal growth and well-being [1–5]. Sabra et al. (2018) found a significant relationship between perceptions of teaching styles and students’ academic engagement, suggesting that supportive teaching practices can enhance students’ psychosocial development and wellness [6]. As engagement increases, students report lower levels of stress and burnout, leading to a more satisfying and productive educational experience [7]. This correlation suggests that fostering academic engagement can lead to a positive feedback loop: higher engagement enhances well-being, which further supports sustained academic involvement. In summary, academic engagement among college students is essential not only for enhancing academic performance but also for promoting personal well-being and fostering a supportive educational community [1,2,5]. Institutions that focus on fostering students’ self-efficacy and self-esteem, offer strong support systems, and adopt teaching practices that promote active participation are likely to see enhanced levels of engagement, which in turn can contribute to greater student persistence and improved academic outcomes [8,9].

Academic engagement, as an important contributor to student success, is shaped by the dynamic interaction of cognitive, motivational, and contextual factors [10–12]. Grounded in expectancy-value theory [10], students’ engagement is shaped not only by their academic values (i.e., perceived importance of education) but also through self-efficacy. Although Self-Determination Theory has identified contributions of different types of motivation [11,12], there is limited understanding of how these factors interact to influence academic engagement. In particular, it remains unclear whether autonomous and controlled motivation function as distinct pathways connecting academic values with engagement. This review integrates relevant theoretical and empirical findings to clarify these links and outlines the specific research gaps addressed in the present study

## 2. Literature review and hypotheses development

### 2.1. Theoretical review

According to Expectancy-Value Theory (EVT), students’ achievement-related choices and persistence are determined by their expectancy beliefs (i.e., beliefs about their competence to succeed) and the subjective value they attach to the task [10].When students endorse stronger academic values, they are more likely to set meaningful learning goals and maintain effort in academic tasks. Academic self-efficacy, which reflects students’ confidence in their academic capabilities, is an important component of expectancy beliefs and further strengthens their willingness to engage in learning activities [10].

While Expectancy–Value Theory explains why students value and expect success in learning, Self-Determination Theory complements this view by addressing the quality and internalization of their motivation. Self-Determination Theory (SDT) emphasizes the quality of motivation by distinguishing between autonomous motivation, which stems from intrinsic interest or personally endorsed values, and controlled motivation, which is driven by external pressures or obligations.Autonomous motivation is believed to foster greater engagement, persistence, and well-being because it supports students’ fundamental needs for autonomy, competence, and relatedness. Conversely, controlled motivation tends to produce more superficial forms of engagement that rely on external rewards or pressures and are less likely to endure over time. Therefore, these two types of motivation are expected to have qualitatively different effects on students’ academic engagement [11,13].

### 2.2. Influence of academic values on academic engagement

The influence of academic values on academic engagement is essential for understanding how students interact with their educational environment [14,15]. Academic values, encompassing personal development, utilitarianism, and responsibility [16], significantly shape students’ motivation, emotional responses, and consequently their level of engagement in academic activities. Personal Development Values emphasize cognitive growth and skill enhancement, which directly impact academic engagement. Students who prioritize their cognitive and competency development tend to engage more deeply with coursework, seeking challenges that expand their knowledge base and refine their abilities. For instance, positive academic emotions can enhance student engagement, suggesting that when students perceive academic tasks as contributing to their personal growth, they are more likely to engage fully [17]. Similarly, positive emotions such as enjoyment and pride strengthen engagement and help build a sense of achievement that supports ongoing academic effort [18]. This aligns with the notion that when values related to competence and growth are prioritized, students are more inclined to invest effort in academic tasks, leading to improved engagement. Utilitarianism in academic values focuses on the practical outcomes of education, such as occupational advancement and material welfare. When students recognized the direct link between their academic efforts and future career opportunities, their engagement increased [19]. This aligns with the findings that understanding the utilitarian benefits of education enhances students’ academic performance through greater engagement, as they perceive their efforts as instrumental to achieving their career and socioeconomic goals [20]. The motivation driven by utilitarian values encourages students to engage more with their studies, as they envision the tangible rewards of their academic endeavors. Responsibility Values, which embody commitments to societal betterment and collective welfare, also play a significant role in academic engagement. Students who develop values reflecting social responsibility tend to engage more with their academic community and actively participate in collaborative learning experiences. However, the relationship between responsibility values and academic engagement is less supported in the literature, particularly in the context referenced by Darensbourg et al. (2013) [21], which did not find a significant relationship between achievement values and engagement in their study. Nevertheless, it remains plausible that students involved in community service projects often report higher levels of academic engagement, as these values reinforce their commitment to societal betterment [21]. In conclusion, the influence of academic values on academic engagement is significant and multifaceted. Values related to personal development, practical outcomes, and social responsibility not only shape students’ motivations but also enhance their emotional responses and commitment to academic activities. Institutions that emphasize the development of these values can increase engagement, with positive effects for both students and the academic community.

### 2.3. Mediation role of academic self-efficacy in the relationship between academic values and academic engagement

The relationships between academic values, academic self-efficacy, and engagement are key factors in shaping students’ experiences within educational settings. Research demonstrated that academic self-efficacy mediates the relationship between academic values and academic engagement [16,22]. First, academic values can significantly enhance students’ academic self-efficacy. When students prioritize their academic values, they are more likely to develop a strong belief in their academic capabilities. For instance, students’ passions for learning can foster self-efficacy, which, in turn, enhances academic engagement [23]. This connection is echoed by Meng & Zhang (2023), who stated that a positive association existed between academic self-efficacy and academic performance, facilitating deeper engagement in academic activities. Academic self-efficacy encourages persistence and informed use of learning strategies, as high self-efficacy leads to improved effort and motivation [24]. Furthermore, academic self-efficacy significantly influences academic engagement [25–28]. Studies indicate that students with higher self-efficacy are more inclined to engage deeply in their studies [29–31]. For instance, students’ self-efficacy is crucial in mediating the effects of teacher support on academic achievement and engagement, highlighting that self-efficacy acts as a motivational force in educational settings [32]. Similarly, there is a significant positive association between academic self-efficacy, academic engagement, and academic achievement, suggesting that increased self-efficacy not only supports engagement but also bolsters academic performance [33]. Additionally, multiple studies have reinforced the intricate relationship between self-efficacy and academic engagement as mediated by the values students hold. For example, academic self-efficacy mediates the relationship between life satisfaction and student engagement, suggesting that positive self-perception can enhance engagement levels through the lens of academic values and personal satisfaction [28]. Moreover, Wu et al. (2020) affirmed the mediating roles of self-efficacy and academic engagement in motivating students, reinforcing the idea that self-efficacy is essential for translating academic values into actionable engagement in learning activities [34]. In conclusion, academic self-efficacy serves as a critical mediator in the relationship between academic values and academic engagement. High academic self-efficacy, spurred by strong academic values, not only enhances students’ motivation and persistence but also fosters a deeper level of engagement in academic tasks [16,22].

### 2.4. Mediation role of autonomous motivation in the relationship between academic values and academic engagement

A growing body of research has identified several important predictors of students’ academic motivation, such as academic values [35], academic resilience [36], mindfulness [37], and gratitude [38]. Among these, academic values are considered a primary driver. Research suggested that strong academic values would promote autonomous motivation [35,39]. For instance, students who understand the meaning and significance of their studies are more likely to show higher levels of intrinsic motivation, thus enhancing their academic achievements [35]. Additionally, students who internalize academic values are more likely to adopt self-motivated learning approaches, which can correlate with higher academic performance, although the study primarily focuses on personality traits [39]. Furthermore, supportive academic environments that emphasize autonomy can enhance the relationship between values and motivation [19]. This finding illustrated the significant influence of external factors, such as teacher autonomy support, in shaping a learning environment that places greater weight on intrinsic rather than extrinsic values, thereby enhancing students’ regulated forms of motivation. On the other hand, while academic values predominantly shape autonomous motivation, they can also influence controlled motivation. For instance, students who feel pressured to meet external academic standards—such as parental expectations or institutional requirements—may engage in learning out of obligation. Autonomous motivation arises when learning activities align with students’ interests, values, or sense of identity, and it is widely regarded as a form of high-quality motivation [11]. Beneficial outcomes (e.g., resilience to cope, need satisfaction, well-being) have been mostly attributed in the literature to the autonomous type of motivation [40,41]. Evidence consistently shows that autonomous motivation fosters sustained and meaningful academic engagement by satisfying students’ basic psychological needs for autonomy, competence, and relatedness [11]. Lu et al. (2022) reported that in supportive classroom contexts, autonomous motivation substantially enhances situational engagement, ultimately leading to more positive academic experiences and outcomes [42].

### 2.5. Mediation role of controlled motivation in the relationship between academic values and academic engagement

Controlled motivation is often less effective than autonomous motivation in promoting long-term academic engagement and success. A study by Datu (2017) highlighted that controlled motivation does not significantly predict academic achievement when compared to intrinsic motivation [43]. This suggests that although academic values can lead to controlled motivations, these motivations are often less beneficial for sustained engagement in learning. Moreover, the notion that individuals with strong academic values may experience mixed motivational states is supported by recent literature. For example, while both intrinsic and extrinsic motivations impact academic performance, intrinsic motivation stemming from valued academic beliefs tends to have more favorable outcomes than controlled motivations driven by external expectations [44]. This dual influence emphasizes the complexity of academic motivation and the need for educators to foster environments that amplify intrinsic values while minimizing pressures that lead students towards controlled forms of motivation. In summary, academic values play a significant role in shaping both autonomous and controlled motivation among students. Strong academic values tend to promote autonomous motivation, facilitating deeper engagement and better academic outcomes, while controlled motivation is often linked to external pressures and may not be conducive to optimal academic success.The relationship between controlled motivation and academic engagement is complex and appears to be context-dependent, as shown in mixed findings from recent studies. According to Self-Determination Theory, controlled motivation, driven by external rewards or internal pressures, is associated with surface learning, stress, and maladaptive outcomes in Western contexts [11]. This theoretical foundation is supported by empirical research of Fan et al. (2024), who found a negative association between controlled motivation and academic adaptation, reinforcing the idea that pressure-driven engagement leads to diminished outcomes [37]. Moreover, negative outcomes (need frustration, ill-being) are often related to controlled motivation [40,41]. Patterns of self-regulatory coping linked to controlled motives further predicted decreased goal progress, increased needs frustration, and decreased needs satisfaction [45].However, this relationship does not hold in all contexts.. Studies by Lu et al. (2022) and López-García (2023) reported positive correlations between controlled motivation and engagement [42,46]. In Lu et al.‘s (2022) study of smart classrooms, the novel and stimulating learning environment might have temporarily enhanced engagement, regardless of the motivation type [42]. Similarly, in López-García’s (2023) study with pre-service Physical Education teachers, the collective and performance-oriented nature of physical education could mean that external pressures are aligned with group goals, thus fostering engagement in the short term [46].

### 2.6. Serial mediation role of academic self-efficacy and autonomous motivation in the relationship between academic values and academic engagement

High levels of academic self-efficacy foster greater engagement in academic activities because students who believe in their abilities are more likely to challenge themselves and pursue their learning goals with sustained effort. For example, Septiana et al. (2021) emphasized that academic self-efficacy is crucial in predicting student engagement in blended learning environments [47]. Autonomous motivation, which encompasses intrinsic motivation and self-determined forms of extrinsic motivation, significantly affects how students respond to academic challenges and the extent to which they engage in their studies. This relationship is partly driven by academic self-efficacy, defined as students’ confidence in accomplishing academic tasks, which subsequently influences their engagement. According to Zhao et al. (2021), the interplay between academic self-efficacy and academic engagement is further influenced by academic passion, which embodies a student’s intrinsic motivation to learn [23]. When students are intrinsically motivated, they are more likely to engage deeply with academic tasks [48–51]. The findings confirm that academic self-efficacy not only prompts students to tackle academic challenges but also fosters an eagerness to learn, thus creating a positive feedback loop that enhances academic engagement. Additionally, academic self-efficacy can significantly impact a student’s intrinsic motivation, which, in turn, predicts engagement levels [27]. Students with high self-efficacy are more likely to adopt a fully engaged learning disposition, characterized by commitment and enthusiasm toward their educational tasks. This reflects how self-efficacy serves as a psychological resource that motivates students to capitalize on their autonomous motivation. The empirical evidence regarding the mediating role of autonomous motivation suggests that it enhances the effectiveness of academic self-efficacy in driving engagement [52,53]. In conclusion, academic self-efficacy and autonomous motivation function as sequential mediators in the relationship between academic values and academic engagement.Students who hold strong academic values are more likely to develop strong academic self-efficacy, which subsequently promotes intrinsic motivation and enhances their engagement.

### 2.7. Serial mediation role of academic self-efficacy and controlled motivation in the relationship between academic values and academic engagement

Controlled motivation, characterized by external pressures or obligations to achieve certain academic outcomes, can influence how students engage with their studies [37,42,46,54]. According to self-determination theory, controlled motivation can potentially weaken the development of intrinsic motivation and autonomous engagement [55]. However, when aligned with academic self-efficacy, controlled motivation may motivate students to engage as it provides external affirmation of their efforts and achievements. Controlled motivation often correlates with increased academic self-efficacy when students feel pressured to meet external expectations [55]. Controlled motivation may mediate this relationship by providing a structured environment wherein self-efficacy beliefs are reinforced through external feedback. Self-efficacy enhances student engagement not only through internal beliefs but also through the motivation to meet external demands [56]. Similarly, students possessing high self-efficacy were more successful at managing the pressures stemming from controlled motivation, leading to improved engagement. Specifically, they noted that when students recognized their capabilities, they became more resilient to external pressures, which prompted increased participation in academic activities [27]. Moreover, the ability to manage emotions affects how students perceive external pressures. Those with strong emotional regulation skills can transform controlled motivation into a positive driving force for engagement, effectively enhancing the relationship between academic self-efficacy and engagement in academic tasks [57]. In conclusion, academic values play an important role in shaping students’ beliefs in their academic capabilities, thereby strengthening their academic self-efficacy. Building on this foundation, controlled motivation plays a mediating role in the relationship between academic self-efficacy and academic engagement.

### 2.8. Hypotheses development

Academic engagement is a crucial factor influencing academic success. Previous studies suggest that students’ values about learning significantly impact their engagement levels. However, the underlying mechanisms explaining this relationship remain underexplored. This study aims to investigate the serial mediation effect of academic self-efficacy and motivation (autonomous motivation and controlled motivation) on the relationship between academic values and academic engagement. Therefore, we proposed the following hypothesis:

H1: Academic self-efficacy mediates the relationship between academic values and academic engagement.

H2: Autonomous motivation mediates the relationship between academic values and academic engagement.

H3: Controlled motivation mediates the relationship between academic values and academic engagement.

H4: Academic self-efficacy, followed by autonomous motivation, forms a serial mediation between academic values and academic engagement.

H5: Academic self-efficacy, followed by controlled motivation, forms a serial mediation between academic values and academic engagement.

**Fig 1:**
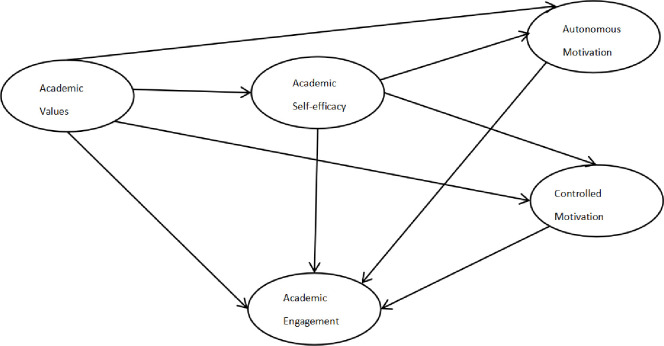
Proposed hypothesized model.

According to the above hypotheses, the current study proposes the following research model (See Fig 1).

## 3. Methods

### 3.1. Research design

This study adopted a quantitative, cross-sectional survey design to examine the relationships among academic values, academic self-efficacy, academic motivation, and academic engagement. Quantitative data were collected using validated self-report questionnaires, and structural equation modeling was employed to test the hypothesized mediation effects.

### 3.2. Participants

A simple random sampling technique was employed to select participants. The sampling frame consisted of college students enrolled in several colleges located in southern China. From these colleges, students were randomly invited to participate, ensuring that each eligible student had an equal chance of selection. We conducted a semPower analysis to determine the necessary sample size. The analysis revealed that a minimum of 676 participants was required to achieve a statistical power of 0.8. Participants for this study were prospectively recruited between November 11th, 2022 and January 5th, 2023. Participants provided written informed consent as part of the survey process. Valid data were collected from 1,110 college students (379 males and 731 females).

### 3.3. Procedures

All procedures involving human subjects received ethical approval from the Ethics Committee for Research Involving Human Subjects at Universiti Putra Malaysia (Protocol Number: JKEUPM-2022–627). Prior to data collection, written consent was obtained from all participants. The survey was conducted in a classroom environment by a trained researcher, who delivered standardized instructions and remained available throughout the session to answer any questions. The questionnaire was distributed and collected online via Wenjuanxing, a widely used survey platform in China. Participation was voluntary and anonymous; no personally identifiable information was collected. Before beginning the survey, respondents were informed of the research purpose, confidentiality assurance, and their right to withdraw at any time. Responses that exhibited straight-line answering patterns, or were completed in an unreasonably short time (less than 2 minutes) were excluded. A total of 1,110 valid responses were retained for analysis.

### 3.4. Measures

#### 3.4.1. Academic values.

In this study, academic values were assessed using the College Students' Academic Values Questionnaire developed by Liao (2010) [16]. The questionnaire includes three dimensions: personal development, utilitarianism, and responsibility, with a total of 19 positive items. Personal development values principally relate to cognitive and competency growth, incorporating domains of knowledge expansion, ability cultivation, and personal refinement. Utilitarianism emphasize instrumental outcomes, particularly occupational advancement, material welfare, and social status attainment. Responsibility values embody prosocial commitments, manifested through dedication to collective welfare and societal betterment.Responses are measured on a 5-point Likert scale, ranging from "strongly disagree" (1) to "strongly agree" (5), with higher scores indicating stronger academic values. The scale demonstrated good reliability and validity, with internal consistency coefficients of 0.88, 0.81, 0.81, and 0.79. Confirmatory factor analysis supported the scale’s structure (χ²/df = 3.59, GFI = 0.92, RMSEA = 0.06, CFI = 0.91, NFI = 0.90).

#### 3.4.2. Academic self-efficacy.

The Academic Self-Efficacy Scale (Liang et al., 2000) was used to assess academic self-efficacy in this study [58]. The scale consists of 22 items and is organized into two dimensions (academic ability self-efficacy and academic behavior self-efficacy), with 11 items per dimension. Among the 11 items in the dimension of academic behavior self-Efficacy, 4 itemsare reverse-scored. Responses are rated on a five-point Likert scale, ranging from “strongly disagree” (1) to “strongly agree” (5). Higher scores indicate a stronger sense of academic self-efficacy. Academic ability self-efficacy refers to an individual’s confidence in their capability to successfully complete academic tasks, achieve good grades, and avoid academic failure. Academic behavior self-efficacy pertains to an individual’s belief in their capacity to employ appropriate learning strategies to attain academic goals. The Cronbach coefficients was 0.82. The results of confirmatory factor analysis showed that χ²/df = 8.709, GFI = 0.922, IFI = 0.931, RMSEA = 0.094, CFI = 0.931, NFI = 0.923. The reliability and validity of the scale is good (Liang, 2000).

#### 3.4.3. Academic motivation.

The instrument used to measure academic motivation is the Academic Motivation Questionnaire which is developed by Liang (2020) [59]. Responses are rated on a seven-point Likert scale, ranging from “totally wrong” (1) to “totally true” (7). The scale consists of 12 positive items, which are divided into two dimensions: autonomous motivation and controlled motivation, in which autonomous motivation includes 5 items and controlled motivation includes 7 items. The Cronbach’s alpha coefficient of the scale were 0.903, 0.790 and 0.882 respectively. The results of confirmatory factor analysis showed that χ²/df = 2.286, GFI = 0.929, IFI = 0.966, RMSEA = 0.076, CFI = 0.965, NFI = 0.940 (Liang, 2020).

#### 3.4.4. Academic engagement.

The instrument used to measure academic engagement is the Academic Engagement Scale (Fang et al., 2008) [60]. The scale was composed of 17 positive items and was scored on a 7-point Likert scale (1 means never, 7 means everyday). The scale consists of three dimensions: vigor, dedication and absorption. Vigor describes students’ capacity to maintain high energy levels and adaptive psychological functioning during study sessions, demonstrating endurance against mental fatigue. Dedication refers to learners’ wholehearted investment in academic pursuits, combining emotional enthusiasm with recognized educational values and task accountability. Absorption represents deep cognitive involvement in learning activities, marked by intrinsic satisfaction and altered time perception during focused engagement. A higher score reflects a greater engagement. The Cronbach coefficients of the whole scale and the sub-factors are 0.951, 0.858, 0.913 and 0.905. The confirmatory factor result is: χ²/ df = 2.77, GFI = 0.86, CFI = 0.93, RMSEA = 0.08, NFI = 0.9, IFI = 0.93 (Fang, 2008). The reliability and validity of the scale is good.

### 3.5. Statistics analysis

Firstly, a descriptive analysis of all variables and a correlation matrix were conducted by SPSS 23.0. Secondly, we adopted the Mplus 8 to analyze the CFAs and perform the structural equation modeling. Finally, bias-corrected bootstrapping with 95% confidence intervals (CIs) [61] was used to test whether these mediating effects were significant.

## 4. Results

### 4.1. Descriptive statistics and correlations

Table 1 presents the means, standard deviations, and Pearson correlation coefficients among the key study variables. Academic values and academic self-efficacy were measured on a 5-point Likert scale, whereas autonomous motivation, controlled motivation, and academic engagement were measured on a 7-point Likert scale. Participants reported moderately high levels of academic values (M = 3.89, SD = 0.669) and self-efficacy (M = 3.37, SD = 0.469), as well as relatively high levels of autonomous motivation (M = 5.51, SD = 1.047). Controlled motivation and academic engagement (M = 4.46, SD = 1.042) were also moderately high (M = 4.87, SD = 0.986). Overall, the correlation results indicate that academic values and self-efficacy are positively and significantly associated with both types of motivation and academic engagement. Notably, autonomous motivation (r = .472, p < .001) showed stronger correlations with academic engagement than controlled motivation (r = .302, p < .001), indicating that it plays a more significant role in fostering students’ active participation in learning.

**Table 1:** Descriptive statistics and correlations.

Variables	Mean	S.D	1	2	3	4
1. academic values	3.89	0.669	1			
2. academic self-efficacy	3.37	0.469	0.567^***^	1		
3. autonomous motivation	5.51	1.047	0.731^***^	0.565^***^	1	
4. controlled motivation	4.87	0.986	0.478^***^	0.404^***^	0.544^***^	1
5. academic engagement	4.46	1.042	0.507^***^	0.662^***^	0.472^***^	0.302^***^

**Note**

* p-value < 0.05

** p-value < 0.01

*** p-value < 0.001.

### 4.2. Hypothesis tests

**Fig 2:**
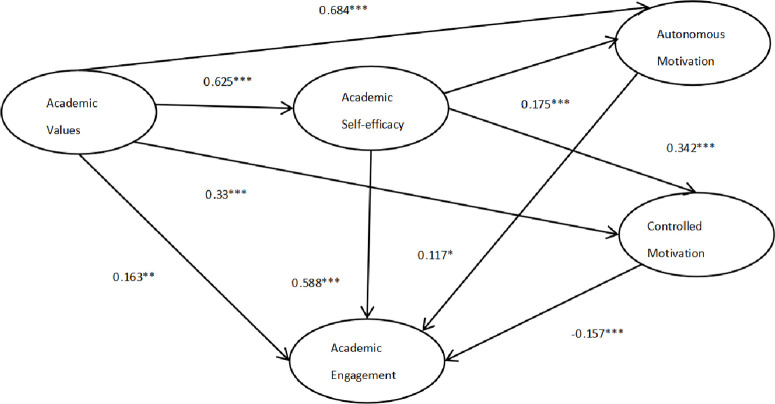
The serial mediating effects model. Note:*p < 0.05; **p < 0.01; ***p < 0.001.

The hypothesized structural equation model demonstrated a good fit to the data: χ2 = 7390.734, df = 2275, χ2/df = 3.249; RMSEA = 0.045, CFI = 0.914, TLI = 0.909, SRMR = 0.054. These fit indices suggest that the proposed model adequately represents the observed data. As illustrated in Fig 2, path coefficients indicate significant associations among academic values, academic self-efficacy, two types of motivation (autonomous and controlled), and academic engagement (Table 2).

**Table 2:** Bias-corrected bootstrap test on the mediating effects.

Paths	Effect	Lower 2.5%	Upper 2.5%	p
values→self-efficacy→engagement	0.367	0.317	0.427	.000
values→autonomous motivation→engagement	0.080	0.013	0.150	.022
values→controlled motivation→engagement	−0.052	−0.086	−0.025	.001
values→self-efficacy→autonomous motivation→engagement	0.013	0.002	0.029	.064
values→self-efficacy→controlled motivation→engagement	−0.033	−0.056	−0.017	.001
Total indirect	0.375	0.287	0.462	.000
Total	0.538	0.471	0.591	.000

A serial multiple mediation analysis was conducted to examine the indirect effects of academic values on academic engagement through academic self-efficacy, autonomous motivation, and controlled motivation. The findings revealed several significant indirect pathways. First, academic values had a significant indirect effect on academic engagement through academic self-efficacy (β = 0.367, 95% CI [0.317, 0.427]), supporting hypothesis 1, indicating that higher academic values were associated with increased academic self-efficacy, which in turn predicted greater academic engagement. In addition, academic values influenced academic engagement through autonomous motivation (β = 0.080, 95% CI [0.013, 0.150]) and controlled motivation (β = −0.052, 95% CI [−0.086, −0.025]), supporting hypothesis 2 and 3. While the pathway through autonomous motivation showed a small but significant positive effect, the indirect effect through controlled motivation was negative, suggesting that autonomous motivation partially enhances the positive impact of academic values on engagement, while higher academic values may reduce engagement when mediated by externally regulated motivation.

Moreover, two significant serial mediation paths involving academic self-efficacy and motivation types were identified, supporting hypothesis 4 and 5. The indirect effect of academic values on academic engagement through academic self-efficacy and subsequently autonomous motivation was positive but small (β = 0.013, 95% CI [0.002, 0.029]). In contrast, the serial path from academic values through academic self-efficacy and controlled motivation to academic engagement was negative (β = −0.033, 95% CI [−0.056, −0.017]), suggesting when academic self-efficacy fosters a more externally regulated form of motivation, it may slightly reduce academic engagement. Taken together, these results reveal the underlying processes by which academic values shape academic engagement, highlighting the serial mediation effects of academic self-efficacy and two motivation types.

Table 3 presents the coefficient of determination (R²). The results indicated that the model explained 39.0% of the variance in academic self-efficacy, 64.8% of the variance in autonomous motivation, 36.7% of the variance in controlled motivation, and 49.7% of the variance in academic engagement. These findings suggest that the proposed model demonstrated acceptable explanatory power, particularly for autonomous motivation and academic engagement, indicating that students’ academic values, self-efficacy, and motivational processes together shape their engagement levels.

**Table 3:** Coefficient of determination (R²).

	Academic Self-efficacy	Autonomous Motivation	Controlled Motivation	Academic Engagement
R²	0.39	0.648	0.367	0.497

Table 4 summarizes the effect size (f²) of each predictor on academic engagement. The results showed that academic values had a negligible effect on academic engagement (f² = 0), suggesting that its direct contribution was minimal after accounting for mediators. In contrast, academic self-efficacy exhibited a large effect (f² = 0.372), indicating that it played a key role in explaining engagement. The effects of autonomous motivation (f² = 0.010) and controlled motivation (f² = 0.034) were small [62], suggesting that although these motivational components influenced engagement, their explanatory power was limited compared with self-efficacy.

**Table 4:** Effect size (f²) of independent and mediating variables on academic engagement.

	Academic Values	Academic Self-efficacy	Autonomous Motivation	Controlled Motivation
f²	0	0.372	0.01	0.034

## 5. Discussion

### 5.1. Mediating role of academic self-efficacy

This finding is consistent with previous research showing that self-efficacy mediates the relationship between values and engagement (β = 0.367, 95% CI [0.317, 0.427]) [16,22]. Academic values strengthen students’ belief in their capabilities, thereby fostering academic self-efficacy. When students prioritize their academic values and hold a genuine passion for learning, they are more likely to develop a strong belief in their academic capabilities [16,22,23]. Higher self-efficacy, in turn, promotes persistence and the effective use of learning strategies, thereby increasing motivation and effort. Hence, students with stronger self-efficacy are more likely to participate actively and meaningfully in their academic work [24–31].

### 5.2. Mediating role of autonomous motivation

The present study revealed that academic values positively influenced academic engagement through autonomous motivation (β = 0.080, 95% CI [0.013, 0.150]). The positive mediation effect supports the assumption that academic values not only directly shape students’ attitudes toward learning but also strengthen their willingness to engage by developing self-determined forms of motivation [35,39]. Moreover, supportive academic environments—particularly those that emphasize autonomy—strengthen this process by encouraging students to prioritize intrinsic values over extrinsic pressures. For example, achievement value and autonomy-supportive teaching practices have been shown to significantly promote engagement through autonomous motivation [19]. Importantly, beneficial outcomes such as resilience, adaptive coping, need satisfaction, and well-being are primarily associated with autonomous motivation, suggesting that students who internalize learning values are more likely to display proactive and self-regulated engagement [17,18,40,41,48–51]. When students are intrinsically motivated, they tend to engage more actively and meaningfully with academic tasks [42], further highlighting autonomous motivation as a mediating role linking academic values to academic engagement.

### 5.3. Mediating role of controlled motivation

The significant negative mediating effect of controlled motivation (β = −0.052) indicates that academic values can indirectly harm academic engagement by fostering a sense of obligation. This aligns with Self-Determination Theory and Western-context studies, which consistently link controlled motivation to surface-level learning and poorer adaptation [11,37]. Empirical evidence further showed that controlled or amotivated forms of regulation were often associated with need frustration and diminished well-being, as well as maladaptive coping patterns that hinder goal attainment and satisfaction [40,41,45].The internal pressure to meet external standards appears to deplete students’ intrinsic energy, leading to diminished engagement. However, this finding contrasts with studies by Lu et al. (2022) and López-García (2023), which reported positive correlations in specific contexts—a smart classroom and physical education, respectively [42,46]. This discrepancy highlights the context-dependent nature of controlled motivation; its negative impact may be neutralized or temporarily reversed in highly stimulating or performance-oriented environments where external pressures align with situational demands. In conclusion, this suggests that promoting autonomous internalization of values is more beneficial for long-term engagement than relying on external pressures.

### 5.4. Serial mediating role of academic self-efficacy and autonomous motivation

The serial mediation effect of academic self-efficacy and autonomous motivation, though statistically significant, was found to be relatively small (β = 0.013). The finding that academic values improve engagement by first building self-efficacy and then boosting internal motivation shows the step-by-step process through which students’ beliefs translate into active learning. As demonstrated by Liao (2010) and Zhang (2018), academic values build a foundational belief in one’s capabilities (academic self-efficacy) [16,22]. In turn, high self-efficacy supports a learning disposition characterized by commitment and enthusiasm, highlighting its role as a psychological resource that facilitates autonomous motivation [27,53]. Consequently, this autonomous motivation leads directly to deeper and more sustained academic engagement [38,42,54].

### 5.5. Serial mediating role of academic self-efficacy and controlled motivation

The serial mediation analysis showing a significant negative indirect effect (β = −0.033, 95% CI [−0.056, −0.017]) reveals a critical mechanism. This path suggests that while academic values foster self-efficacy [16,22], this enhanced confidence does not necessarily lead to positive engagement. Instead, it can sometimes heighten a student’s sensitivity to external pressures, thereby strengthening controlled motivation [55]. When high self-efficacy aligns with controlled motivation, driven by external rewards rather than inherent interest, it can compromise constructive engagement [57]. Consequently, the initial positive influence of academic values is partially undermined, resulting in a negative effect on the quality or sustainability of academic engagement.

## 6. Limitations and implications

This study sheds light on the pathways that shape academic engagement among Chinese university students, emphasizing the distinct contributions of academic value orientations, self-efficacy beliefs, autonomous motivation, and controlled motivation. However, some limitations should be acknowledged. First, the reliance on self-report questionnaires raises concerns about potential biases, such as social desirability bias or inaccurate self-perceptions. Future investigations could adopt multi-source data or behavioral measures to improve measurement validity. Second, as the participants were recruited from southern China, the findings may not be fully generalizable to students in other geographical or cultural contexts. Broader sampling across diverse regions and educational contexts would enhance external validity. Third, the cross-sectional nature of the study restricts causal interpretations. To explore motivational processes over time, future research should consider longitudinal or experimental designs. Lastly, while this study centered on individual-level determinants, future work could be enriched by examining contextual factors—such as teacher support, peer dynamics, and institutional environment—to provide a more in-depth understanding of academic engagement.

The results of this study carry several educational implications regarding how academic values influence students’ engagement through motivational and psychological mechanisms. First, the strong and significant indirect path from academic values to academic engagement through academic self-efficacy highlights the critical role of self-efficacy in turning students’ internalized academic beliefs into active learning behaviors. This suggests that efforts to foster students’ belief in the value of learning must be accompanied by strategies that enhance their confidence in their academic abilities. Educational interventions such as mastery-based learning, self-reflection exercises, and personalized feedback could be particularly effective in this regard. Second, the mediating role of autonomous and controlled motivation provides detailed insights. The positive indirect effect of academic values on engagement through autonomous motivation suggests that when students perceive their studies as valuable, they are more likely to develop self-determined forms of motivation, which in turn fosters deeper engagement. Educators should therefore create autonomy-supportive learning environments that reinforce the intrinsic value of academic tasks and provide students with opportunities for choice and self-direction. Conversely, the negative indirect effect through controlled motivation indicates that when academic values give rise to motivation rooted in external pressure or obligation, students’ engagement may be weakened. This highlights the potential risks of over-relying on extrinsic incentives or normative pressures to promote learning, even when students possess strong academic values. Educational policies and classroom practices should thus avoid equating high achievement expectations with sustainable motivation, especially if they threaten students’ sense of autonomy. Finally, the serial mediation effects further highlight the complexity of motivational processes. The small but significant indirect pathway through academic self-efficacy and autonomous motivation highlights the combined benefit of enhancing both students’ self-beliefs and their internalized motivation. In contrast, the negative serial effect through self-efficacy and controlled motivation suggests that even confident students may disengage if their motivation is primarily driven by external pressures. These findings point to the need for integrated educational practices that build both competence and autonomy to maximize student engagement in meaningful and sustainable ways.

## 7. Conclusions

This study provides new insights into our understanding of the dynamic relationships between academic values, self-efficacy, motivation, and engagement in higher education. Drawing on a large sample of Chinese college students, the findings highlight that both academic values and self-efficacy show significant direct effects on academic engagement, while also influencing it indirectly through distinct motivational pathways. Specifically, autonomous motivation emerged as a positive mediator, linking values and self-efficacy to engagement. In contrast, controlled motivation showed a weaker yet significant mediating role, demonstrating a negative effect on engagement. These results support the theoretical distinction between autonomous and controlled forms of motivation, and emphasize the importance of fostering value-driven and efficacy-promotinglearning environments to promote meaningful student engagement. Overall, the study offers empirical evidence for a complex motivational mechanism and calls for educational interventions that strengthen internalized values and academic self-beliefs to enhance student engagement.
